# Association between preoperative sarcopenia and prognosis of pancreatic cancer after curative-intent surgery: a updated systematic review and meta-analysis

**DOI:** 10.1186/s12957-024-03310-y

**Published:** 2024-01-30

**Authors:** Chenming Liu, Liang An, Siyuan Zhang, Shiqing Deng, Neng Wang, Haijun Tang

**Affiliations:** 1 Department of Hepatopancreatobiliary Surgery, Shaoxing People’s Hospital, Shaoxing, Zhejiang China; 2 Department of General Surgery, Sir Run Run Shaw Hospital, School of Medicine, Zhejiang University, Hangzhou, Zhejiang China; 3 Department of Gastrointestinal Surgery, Shaoxing People’s Hospital, Shaoxing, Zhejiang China; 4 Department of Hepatopancreatobiliary Surgery, The First Affiliated Hospital of Jinzhou Medical University, Jinzhou, Liaoning China; 5 Department of Breast and Thyroid Surgery, General Hospital of Huainan Eastern Hospital Group, Huainan, Anhui China; 6 Department of Hepatopancreatobiliary Surgery, The Quzhou Affiliated Hospital of Wenzhou Medical University, Quzhou People’s Hospital, Quzhou, Zhejiang China

**Keywords:** Sarcopenia, Pancreatic neoplasm, Prognosis, Meta-analysis

## Abstract

**Background:**

Sarcopenia is associated with poor outcomes in many malignancies. However, the relationship between sarcopenia and the prognosis of pancreatic cancer has not been well understood. The aim of this meta-analysis was to identify the prognostic value of preoperative sarcopenia in patients with pancreatic cancer after curative-intent surgery.

**Methods:**

Database from PubMed, Embase, and Web of Science were searched from its inception to July 2023. The primary outcomes were overall survival (OS), progression-free survival (PFS), and the incidence of major complications. The hazard ratio (HR), odds ratio (OR), and 95% confidence intervals (CIs) were used to assess the relationship between preoperative sarcopenia and the prognosis of patients with pancreatic cancer. All statistical analyses were conducted by Review Manager 5.3 and STATA 17.0 software.

**Results:**

A total of 23 retrospective studies involving 5888 patients were included in this meta-analysis. The pooled results demonstrated that sarcopenia was significantly associated with worse OS (HR = 1.53, *P* < 0.00001) and PFS (HR = 1.55, *P* < 0.00001). However, this association was not obvious in regard to the incidence of major complications (OR = 1.33, *P* = 0.11).

**Conclusion:**

Preoperative sarcopenia was preliminarily proved to be associated with the terrible prognosis of pancreatic cancer after surgery. However, this relationship needs to be further validated in more prospective studies.

**Supplementary Information:**

The online version contains supplementary material available at 10.1186/s12957-024-03310-y.

## Introduction

Pancreatic cancer is a highly malignant solid tumor with 5-year survival rate less than 10% [1, 2]. In recent years, its incidence and mortality are still gradually increasing, and it is predicted to be the second leading cause of cancer-related death in the USA by 2030 [3]. Although the application of systemic chemotherapy and targeted therapy has greatly benefited patients with pancreatic cancer in recent years, surgery remains the only curative-intent treatment strategy. However, postoperative survival rate is still unsatisfactory due to its large probability of recurrence and metastasis [4, 5]. Previous studies on prognosis following pancreatectomy have mainly focused on tumor-specific factors such as tumor’s differentiation, perivascular invasion, and lymph node invasion [6–8]. However, their predictive abilities were skeptical due to the instability of these indicators.

In recent years, there has been increasing interest in the association between body composition and prognosis due to its simplicity and practicality. Sarcopenia, referring to age-dependent reduction in skeletal muscle volume, was first described in 1989 [9]. Sarcopenia was a kind of progressive and widespread skeletal muscle disease associated with an increased likelihood of adverse outcomes, including falls, fractures, physical disability, and death [10]. It has been found to be a potential risk factor for morbidity and mortality in patients with gastrointestinal malignancies [11].

Most patients with pancreatic cancer were prone to skeletal muscle depletion, leading to reduced tolerance for postoperative adjuvant therapy [12, 13]. Several recent studies have attempted to investigate the effect of sarcopenia on the prognosis of pancreatic cancer, but the outcomes of these studies have been more or less controversial [14–17]. Evidence needs to be updated, so the aim of this systematic review and meta-analysis is to clarify the relationship between preoperative sarcopenia and the prognosis of pancreatic cancer.

## Materials and methods

The systematic review and meta-analysis followed Preferred Reporting Items for Systematic Review and Meta-Analysis (PRISMA) guidelines [18]. The registration number was INPLASY202390060. The protocol could be found in Inplasy Protocol 5298 – INPLASY.

### Literature search strategy

Two independent reviewers searched PubMed, Embase, and Web of science from its inception to July 2023. The language of search results was limited to English. Subsequently, the two persons checked each other and tried to reach a consensus. The detailed search strategies are presented in the Additional file 1.

### Inclusion and exclusion criteria

Inclusion criteria were as follows: patients were pathologically diagnosed with pancreatic cancer; sarcopenia was evaluated by cross-sectional computed tomography (CT) scan of the third lumbar (L3) vertebra with respective cut-off values defined by sex before surgery; the measurement method of sarcopenia included skeletal muscle index (SMI) and psoas muscle index (PMI), as described in previous studies [19, 20], which represented two most common measurement methods; the definition of cut-off values included various standards, such as receiver operating characteristic (ROC) curves, Martin’s definition [21], Prado’s definition [22], and lowest quantile; outcomes were evaluated by prognostic indicators such as overall survival (OS) and/or progression-free survival (PFS) and the incidence of postoperative complications.

Exclusion criteria were as follows: patients were pathologically diagnosed as benign or borderline pancreatic tumors; sarcopenia was assessed by methods other than CT, such as bioelectrical analysis (BIA) and dual-energy X-ray absorptiometry (DXA); the cut-off values for sarcopenia were not clearly defined; the types of studies were conference abstracts, case reports, letters, and reviews; the time to evaluate sarcopenia took place postoperatively or the treatment strategy was palliative.

### Outcomes

The primary outcomes were OS, PFS, and the incidence of major complications (grade III–IV) according to the Clavien-Dindo classification [23]. Secondary outcomes were the incidence of overall complications (grade I–IV) according to the Clavien-Dindo classification, as well as surgery-specific complications including clinically relevant postoperative pancreatic fistula (CR-POPF), post-pancreatectomy hemorrhage (PPH), delayed gastric empty (DGE), and surgical site infection (SSI) [24–26].

### Data extraction

Two investigators independently extracted the following information from each study: publishing year, the name of first author, country, sample size, perioperative treatment (including neoadjuvant and adjuvant therapy), the measurement approach of sarcopenia, the cut-off values for sarcopenia, and clinical outcomes.

### Assessment of methodological quality

Two independent investigators assessed the quality of the included studies on the Newcastle–Ottawa Scale (NOS) [27]. The contents of the scale included case selection, cohort comparison, and exposure risk assessment. Only studies with NOS score of six or higher were included in the final meta-analysis.

### Statistical analysis

Survival data were evaluated by hazard ratio (HR) and their 95% corresponding intervals (CIs) in multivariate regression analysis, and categorical variables by odds ratio (OR). The Cochrane’s *Q*-test and *I*^2^ statistics were used to assess statistical heterogeneity. The cut-off value of low, moderate, and high heterogeneity was 25%, 50% and 75%, respectively. When the value of total heterogeneity exceeded 50%, we used the random-effect model. Otherwise, the fixed-effect model was applied. Subgroup analyses stratified by measurement approach of sarcopenia (SMI or PMI), region of studies (Asia or non-Asia), and definition of cut-off values (ROC curve, Martin’s definition, Prado’s definition, and lowest quantile) were performed further to find out the source of heterogeneity. *P* < 0.05 was regarded as statistically significant. In order to explore the possibility of publication bias, we applied funnel plots and Egger’s test. All analyses were conducted by Review Manager 5.3 software (Copenhagen: The Nordic Cochrane Centre, The Collaboration, 2011) and STATA 17.0 software (College Station, TX).

## Results

### Study selection

**Fig. 1:**
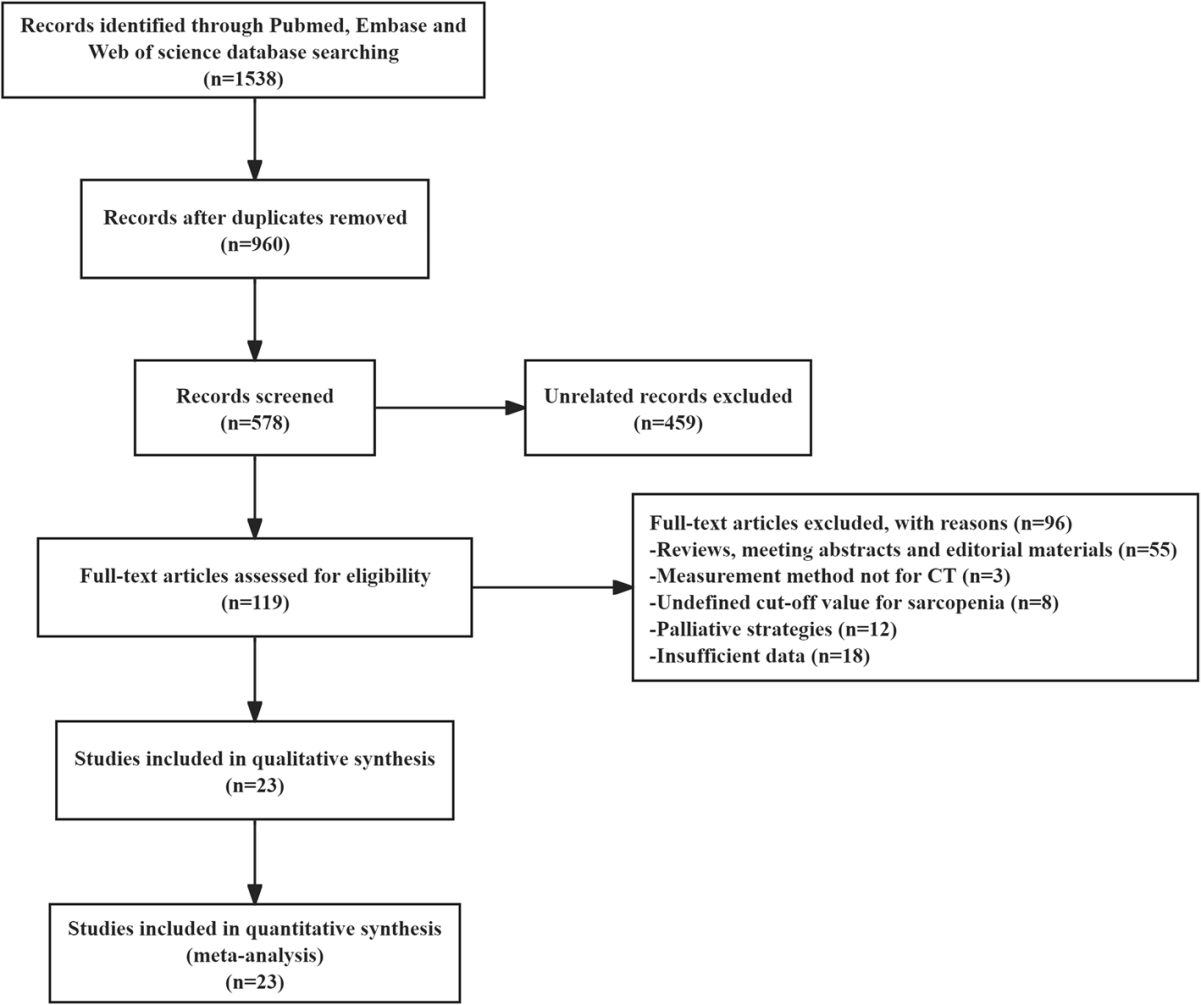
Flow diagram of included studies

We searched 1538 articles from the electronic databases (PubMed, Embase, and Web of Science). After removing duplicates and unrelated studies, 119 full-text studies were assessed for eligibility. Eventually, 23 studies were eligible for qualitative synthesis after careful examination [14–17, 28–46]. The detailed flow diagram is shown in Fig. 1.

### Basic characteristics of included studies

A total of 5888 patients with pancreatic cancer were incorporated into our meta-analysis. Publication year of studies ranged from 2012 to 2023. Seventeen (73.9%) studies were from Asian countries and only 6 (26.1%) from non-Asian countries. The majority of studies applied SMI to measure sarcopenia. And the definition of sex-related cut-off values for sarcopenia included 5 approaches, ROC curves (30.4%), Martin’s definition (13.0%), Prado’s definition (21.7%), Contal-O’Quigley method (4.3%), and lowest quantile (30.6%). The detailed information is listed in Table 1.

**Table 1:** Basic characteristics of included studies

Year	Author	Country	Sample size	Neoadjuvant treatment	Adjuvant treatment	Measurement	Cut-off value	Primary outcome	Secondary outcome
2023	Shen [46]	China	614	NA	379 (61.7%)	SMI	Male < 52.4cm^2^/m^2^; female < 38.5 cm^2^/m^2^	OS (HR = 1.23, 95% CI 0.93–1.62) MC	NA
2022	Özkul [45]	Turkey	115	NA	NA	SMI	Male < 56.44cm^2^/m^2^; female < 43.56cm^2^/m^2^	OS (HR = 1.23, 95% CI 1.33–1.85)	NA
2022	Cai [44]	China	115	NA	84 (73.0%)	SMI	Male < 45.16cm^2^/m^2^; female < 34.65cm^2^/m^2^	OS (HR = 2.31, 95% CI 1.21–4.40) PFS (HR = 1.91, 95% CI 1.15–3.17)	NA
2022	Kim [14]	Korea	347	0(0.0%)	226 (65.1%)	SMI	Following legends behind^a^	OS (HR = 1.40, 95% CI 0.93–2.10) PFS (HR = 1.43, 95% CI 0.96–2.13)	NA
2021	Rom [15]	Israel	111	3(2.7%)	73 (65.8%)	SMI	Male < 44.35cm^2^/m^2^; female < 34.82cm^2^/m^2^	OS (HR = 1.73, 95% CI 1.07–2.80) MC	OC
2021	d’Engremont [43]	France	76	NA	NA	SMI	Male < 52.4cm^2^/m^2^; female < 38.5cm^2^/m^2^	PFS (HR = 1.78, 95% CI 1.01–3.14)	NA
2020	Ryu [41]	Korea	548	22(4.0%)	NA	SMI	Male < 50.18cm^2^/m^2^; female < 38.63cm^2^/m^2^	OS (HR = 1.16, 95% CI 0.92–1.46) MC	CR-POPF PPH DGE SSI
2020	Xu [42]	China	152	NA	NA	PMI	Male < 4.78cm^2^/m^2^; female < 3.46cm^2^/m^2^	MC	NA
2020	Peng [40]	China	116	3 (2.6%)	58 (34.9%)	SMI	Male < 42.2cm^2^/m^2^; female < 33.9cm^2^/m^2^	OS (HR = 2.51, 95% CI 1.03–6.12) PFS (HR = 1.00, 95% CI 0.55–1.81) MC	NA
2019	Ratnayake [39]	New Zealand	89	NA	NA	SMI	Male < 43cm^2^/m^2^ (BMI < 25 kg/m^2^), 53cm^2^/m^2^ (BMI > 25 kg/m^2^); female < 41cm^2^/m^2^	MC	OC CR-POPF PPH DGE SSI
2019	Gruber [38]	Austria	133	20 (15.0%)	NA	SMI	Male < 52.4cm^2^/m^2^; female < 38.5cm^2^/m^2^	OS (HR = 1.51, 95% CI 1.04–2.19) MC	CR-POPF
2018	Yamane [37]	Japan	99	NA	NA	SMI	Male < 43cm^2^/m^2^ (BMI < 25 kg/m^2^), 53cm^2^/m^2^ (BMI > 25 kg/m^2^); female < 41cm^2^/m^2^	NA	CR-POPF
2018	Wagner [36]	Austria	424	NA	NA	PMI	Male < 20.74cm^2^/m^2^; female < 14.65cm^2^/m^2^	MC	OC
2018	Tankel [35]	Israel	61	3 (4.9%)	NA	PMI	Male < 4.92cm^2^/m^2^; female < 3.62cm^2^/m^2^	MC	CR-POPF DGE
2018	EI Amrani [34]	France	107	NA	NA	SMI	Male < 52.4cm^2^/m^2^; female < 38.5cm^2^/m^2^	OS (HR = 2.04, 95% CI 0.93–4.47) MC	CR-POPF DGE SSI
2018	Choi [16]	Korea	180	NA	NA	SMI	Male < 45.3cm^2^/m^2^; female < 39.3cm^2^/m^2^	OS (HR = 1.78, 95% CI 1.20–2.65) MC	OC
2017	Takagi [33]	Japan	219	NA	NA	SMI	Male < 68.5cm^2^/m^2^; female < 52.5cm^2^/m^2^	MC	CR-POPF DGE SSI
2017	Okumura [32]	Japan	301	33 (11.0%)	216 (71.2%)	SMI	Male < 47.1cm^2^/m^2^; female < 36.6cm^2^/m^2^	OS (HR = 1.79, 95% CI 1.24–2.58) PFS (HR = 1.60, 95% CI 1.18–2.16) MC	CR-POPF
2017	Ninomiya [17]	Japan	265	0 (0.0%)	174 (65.6%)	SMI	Male < 43.75cm^2^/m^2^; female < 38.5cm^2^/m^2^	OS (HR = 2.11, 95% CI 1.20–3.70) MC	NA
2016	Nishida [31]	Japan	266	22 (8.3%)	NA	SMI	Male < 43cm^2^/m^2^ (BMI < 25 kg/m^2^), 53cm^2^/m^2^ (BMI > 25 kg/m^2^); female < 41cm^2^/m^2^	MC	CR-POPF DGE SSI
2015	Okumura [30]	Japan	230	24 (10.4%)	NA	PMI	Male < 5.90cm^2^/m^2^; female < 4.07cm^2^/m^2^	OS (HR = 1.99, 95% CI 1.37–2.90) PFS (HR = 1.60, 95% CI 1.14–2.24) MC	NA
2015	Amini [29]	USA	763	NA	NA	PMI	Male < 5.64cm^2^/m^2^; female < 4.15cm^2^/m^2^	OS (HR = 1.46, 95% CI 1.11–1.92) MC	OC
2012	Peng [28]	China	557	NA	NA	PMI	Male < 4.92cm^2^/m^2^; female < 3.62cm^2^/m^2^	OS (HR = 1.63, 95% CI 1.28–2.08) MC	OC

*SMI* Skeletal muscle index, *PMI* Psoas muscle index, *BMI* Body mass index, *HR* Hazard ratio, *CI* Confidential interval, *OS* Overall survival, *PFS* Progression-free survival, *MC* Major complications, *OC* Overall complications, *CR-POPF* Clinically related postoperative pancreatic fistula, *PPH* Post-pancreatectomy hemorrhage, *DGE* Delayed gastric empty, *SSI* Surgical site infection, *NA* No available

BMI < 23 kg/m^2^, age < 65 years: male < 45.25 cm^2^/m^2^, female < 37.39 cm^2^/m^2^; BMI < 23 kg/m^2^, age ≥ 65 years: male < 48.86 cm^2^/m^2^, female < 38.85 cm^2^/m^2^

BMI ≥ 23 kg/m^2^, age < 65 years: male < 54.89 cm^2^/m^2^, female < 44.90 cm^2^/m^2^; BMI ≥ 23 kg/m^2^, age ≥ 65 years: male < 49.66 cm^2^/m^2^, female < 49.84 cm^2^/m^2^

^a^Kim et al.

### Primary outcomes

#### The relationship between preoperative sarcopenia and OS

**Fig. 2:**
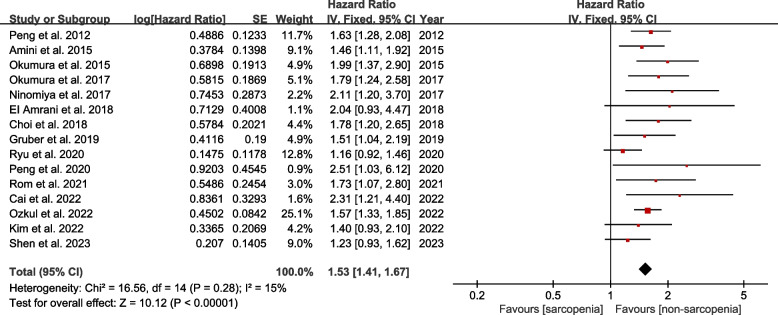
Forest plot of comparison in overall survival between sarcopenia and non-sarcopenia

The impact of preoperative sarcopenia on OS was explored in fifteen studies. The pooled HR demonstrated that preoperative sarcopenia was significantly associated with worse OS (HR = 1.53, 95% CI 1.41–1.67, *P* < 0.00001; *I*^2^ = 15%, *P* = 0.28) (Fig. 2). Subgroup analyses based on the measurement approach, region of studies, and different definitions of cut-off values confirmed the similar results (all *P* < 0.05). And all heterogeneity was moderate or low (Table 2).

**Table 2:** Subgroup analysis for overall survival

	No. studies	Samples	HR	95% CI	*P* value	*I*^2^
Measurement method						
SMI	12	2952	1.49	1.36–1.63	< 0.00001	21%
PMI	3	1550	1.62	1.38–1.91	< 0.00001	0%
Region of studies						
Asia	12	3499	1.54	1.41–1.68	< 0.00001	31%
Non-Asia	3	1003	1.51	1.22–1.86	0.0001	0%
Definition of cut-off values						
ROC curves	5	877	1.69	1.48–1.94	< 0.00001	0%
Lowest quantile	5	2159	1.45	1.27–1.65	< 0.00001	34%
Prado’s definition	4	1119	1.44	1.18–1.76	0.0003	22%

*SMI* Skeletal mass index, *PMI* Psoas mass index, *HR* Hazard ratio, *CI* Corresponding intervals

#### The relationship between preoperative sarcopenia and PFS

**Fig. 3:**
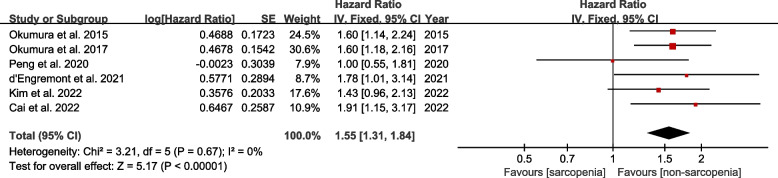
Forest plot of comparison in progression-free survival between sarcopenia and non-sarcopenia

Six studies evaluated the association between preoperative sarcopenia and PFS. The pooled HR showed that preoperative sarcopenia was strongly related to worse PFS (HR = 1.55, 95% CI 1.31–1.84, *P* < 0.00001; *I*^2^ = 0%, *P* = 0.67) (Fig. 3). However, we were not able to further perform subgroup analysis due to the limited available information.

#### The relationship between preoperative sarcopenia and the incidence of major complications

**Fig. 4:**
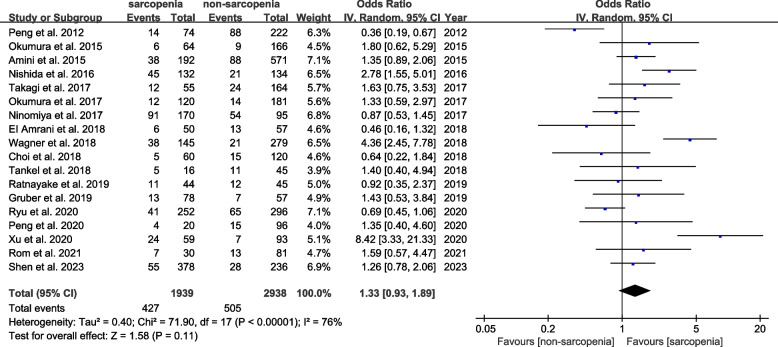
Forest plot of comparison in the incidence of major complications between sarcopenia and non-sarcopenia

**Fig. 5:**
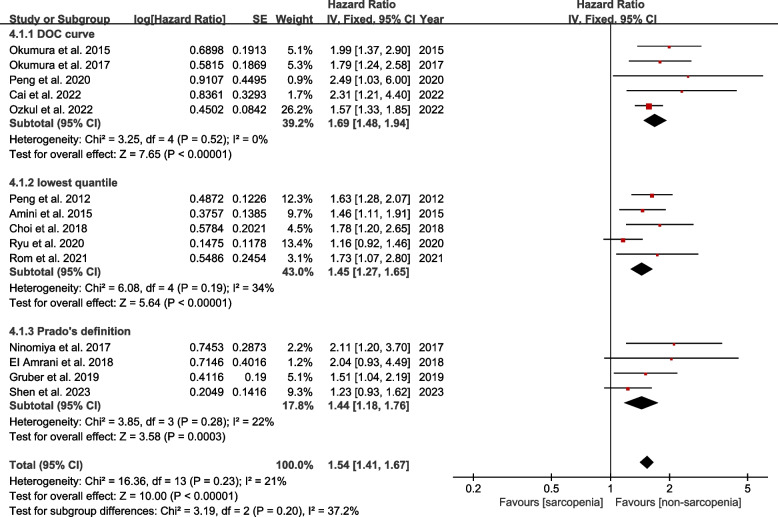
Forest plot of comparison in the incidence of major complications between sarcopenia and non-sarcopenia according to different definitions of cut-off values

Eighteen studies including 4877 participants explored the predictive role of preoperative sarcopenia for major complications. Contrary to OS and PFS, preoperative sarcopenia was not obviously associated with high incidence of major complications (OR = 1.33, 95% CI 0.93–1.89, *P* = 0.11; *I*^2^ = 76%, *P* < 0.00001) (Fig. 4). However, interestingly, subgroup analysis stratified by the different definitions of cut-off values showed the inconsistent results. The pooled OR of those studies whose cut-off values were defined by ROC curves demonstrated preoperative sarcopenia’s strong relevance to the increased incidence of major complications (OR = 2.73, 95% CI 1.35–5.53, *P* = 0.005; *I*^2^ = 0%, *P* = 0.01), but this relevance was not shown in studies defined by the other three definitions (Fig. 5, Table 3).

**Table 3:** Subgroup analysis for the incidence of major complications

	No. studies	Samples	OR	95% CI	*P* value	*I*^2^
Measurement method						
SMI	12	2951	1.13	0.84–1.53	0.40	47%
PMI	6	1926	1.86	0.77–4.51	0.17	89%
Region of studies						
Asia	13	3359	1.30	0.86–1.97	0.21	76%
Non-Asia	5	1518	1.39	0.68–2.82	0.37	79%
Definition of cut-off values						
ROC curves	5	1223	2.73	1.35–5.53	0.005	69%
Lowest quantile	7	2178	0.92	0.59–1.45	0.73	66%
Martin’s definition	2	355	1.70	0.58–5.02	0.33	74%
Prado’s definition	4	1121	1.00	0.69–1.45	1.00	19%

*SMI* Skeletal mass index, *PMI* Psoas mass index, *OR* Odds ratio, *CI* Corresponding intervals

### Secondary outcomes

#### Overall and surgery-related complications

Impact of preoperative sarcopenia on overall complications was reported in six studies. Preoperative sarcopenia was not obviously related to the increased incidence of postoperative overall complications (OR = 1.33, 95% CI 0.84–2.12, *P* = 0.23; *I*^2^ = 75%, *P* = 0.001). And the increased probability of surgery-related complications, including CR-POPF, PPH, DGE, and SSI, was not observed to have a strong association with preoperative sarcopenia, either (all *P* > 0.05) (Additional file 2). And the results of subgroup analyses were consistent (Tables 4 and 5).

**Table 4:** Subgroup analysis for the incidence of overall complications

	No. studies	Samples	OR	95% CI	*P* value	*I*^2^
Measurement method						
SMI	3	380	1.29	0.82–2.03	0.27	0%
PMI	3	1483	1.34	0.62–2.87	0.45	75%
Region of studies						
Asia	3	587	1.10	0.66–1.86	0.71	43%
Non-Asia	3	1276	1.49	0.69–3.23	0.31	86%

*SMI* Skeletal mass index, *PMI* Psoas mass index, *OR* Odds ratio, *CI* Corresponding intervals

**Table 5:** Subgroup analysis for the incidence of surgical related complications

	No. studies	Samples	OR	95% CI	*P* value	*I*^2^
The incidence of CR-POPF	8	1522	0.97	0.65–1.44	0.87	42%
Region of studies						
Asia	5	1193	0.98	0.54–1.77	0.93	63%
Non-Asia	3	329	0.87	0.50–1.50	0.61	0%
The incidence of DGE	6	1290	1.18	0.67–2.08	0.56	54%
Region of studies						
Asia	4	1094	1.26	0.53–2.99	0.60	69%
Non-Asia	2	196	1.13	0.57–2.25	0.73	13%
The incidence of SSI	5	1229	1.31	0.75–2.29	0.34	60%
Region of studies						
Asia	3	1033	1.53	0.63–3.72	0.35	77%
Non-Asia	2	196	1.04	0.57–1.90	0.91	0%

*OR* Odds ratio, *CI* Corresponding intervals, *CR-POPF* Clinically related postoperative pancreatic fistula, *DGE* Delayed gastric empty, *SSI* Surgical site infection

#### Publication bias

The symmetrical distribution of funnel plots showed no significant risk of publication bias (Additional file 3). Moreover, Egger’s regression test suggested that publication bias was insignificant for OS (*P* = 0.757), PFS (*P* = 0.684), and the incidence of major complications (*P* = 0.448).

## Discussion

We conducted a systematic review and meta-analysis of 23 studies to investigate the relationship between preoperative sarcopenia and the prognosis of pancreatic cancer after radical surgery, including OS, PFS, and the incidence of complications (overall complications and major complications, as well as four surgical-related complications including CR-POPF, PPH, DGE, and SSI). Our results were encouraging, suggesting that preoperative sarcopenia significantly reduced survival time (OS and PFS). However, our analysis did not confirm that sarcopenia was strongly associated with high incidence of postoperative complications.

Basically consistent with our results, the first meta-analysis conducted by Mintziras et al. in patients with pancreatic ductal adenocarcinoma confirmed that sarcopenia was strongly associated with worse OS (HR = 1.49, 95% CI 1.27–1.74, *P* < 0.001) [47]. However, they did not exclude those patients with palliative treatment. Moreover, analyses of the incidence of major complications and CR-POPF in sarcopenia were not performed due to limited data. Bundred et al. showed that sarcopenia was not significantly associated with the incidence of postoperative complications or CR-POPF [48]. However, of the studies they included, only five and two, respectively, reported the incidence of major complications and CR-POPF. In addition, the generalization of their results was limited by the high heterogeneity caused by non-standardized measurement methods, such as BIA and DXA. CT could make up for the unavoidable disadvantage of BIA and DXA to patients caused by repeated doses of radiation, and studies have confirmed that CT scan has been shown to be more sensitive to small changes in muscle area than DXA [49, 50]. So, based on the recent consensus from the European Working Group on Sarcopenia in Older People and the Asian Working Group for Sarcopenia, CT imaging at the level of the L3 vertebra represents a standardized method to quantify the skeletal musculature [51, 52]. Thormann et al. concluded that sarcopenia was strongly relevant to dismal prognosis in both radical and palliative settings. Unfortunately, they did not conduct further subgroup analyses to explore the sources of heterogeneity [53].

The mechanism of the association between sarcopenia and poor prognosis has not been well understood. Sarcopenia is not merely a loss of muscle mass or quantity, but a disorder that reflects a disorder of immune nutritional status, and its relationship with the tumor micro-environment is still being studied [54]. Several nutritional and immune factors were found to have an important role in people with sarcopenia. Previous studies have reported that high neutrophil–lymphocyte ratio (NLR) was an independent indicator of muscle mass loss [45]. A recent meta-analysis showed that in patients with pancreatic cancer, lower NLR had better OS and PFS in patients with pancreatic cancer [55]. In addition, several studies have demonstrated that sarcopenia was associated with insulin resistance, vitamin D deficiency, elevated levels of inflammatory cytokines (such as tumor necrosis factor-alpha and interleukin-6), and decreased concentrations of muscle factors (such as interleukin-15) [56–58]. Under the action of the above factors, the body’s immune system is weakened, and the postoperative wound healing is poor, thus affecting the risk of postoperative complications.

Since sarcopenia is associated with unsatisfactory postoperative survival rate and high incidence of complications, perioperative intervention is important to reduce these risks. Nutritional counseling and oral nutritional supplements may also be available as intervention options for the treatment of cachexia [59, 60]. Studies have shown that in patients with gastric cancer, preoperative exercise and nutritional support programs can reduce the incidence of sarcopenia and improve postoperative outcomes [61].

To analyze the sources of heterogeneity, we performed subgroup analyses by regions of studies (Asian or non-Asian), measurement methods of sarcopenia (SMI or PMI), and definition criteria for sex-specific cut-off values, respectively. Our subgroup analyses of different study regions and measurement methods did not change the overall results. But interestingly, our research showed that under the criteria of cut-off values defined by the ROC curve, preoperative sarcopenia was strongly associated with worse OS (HR = 1.69, 95% CI 1.48–1.94, *P* < 0.00001) and higher incidence of complications (OR = 2.73, 95% CI 1.35–5.53, *P* = 0.005). In contrast, the relationship was less significant or non-significant based on the criteria of other definitions, such as the lowest quantile, Prado’s, and Martin’s definition. We speculate that this phenomenon may be related to the objectivity and accuracy of ROC curve based on the data itself, free from external interference. Therefore, this finding may provide a novel direction for more accurate definition of cut-off values for sarcopenia in the future. However, at present, no unanimously accepted cut-off values have been established for CT-based sarcopenia in Asian populations. Therefore, more large-scale studies are needed in the future to establish standardized cut-off values for sarcopenia in different populations and confirm these observations.

We have to admit that our study has several limitations. First, all the studies we included were retrospective cohort studies. In the future, large-scale randomized controlled trials are needed to further clarify the relationship between sarcopenia and the prognosis of pancreatic cancer. Second, due to the limited information available from the included studies, we did not conduct more subgroup analyses of other important indicators that may influence prognosis, such as tumor’s stage, gender, perioperative treatment (including neoadjuvant and adjuvant therapy), and surgical procedure. Finally, we did not analyze biomarkers that might affect muscle quality, such as fat infiltration and accumulation, because relevant studies were still insufficient. Sarcopenia reflects a combination of muscle quantity and mass. However, to the best of our knowledge, this study is the first meta-analysis to analyze the relationship between sarcopenia and the prognosis after radical resection of pancreatic cancer according to different definition criteria of sarcopenia cut-off values, which may provide novel direction for accurate exploration in the future.

## Conclusion

Preoperative sarcopenia was preliminarily proved to be significantly associated with the poor prognosis of pancreatic cancer patients after radical surgery. However, this relationship needs to be further validated in more prospective studies.

### Supplementary Information

**Additional file 1.**

**Additional file 2: Supplementary Figure 1.** Forest plots of comparison between sarcopenia and non-sarcopenia. (A) overall complications, (B) CR-POPF, (C) PPH, (D) DGE, (E) SSI.

**Additional file 3: Supplementary Figure 2.** Funnel plots for examination of publication bias. (A) overall survival, (B) major complications, (C) profession-free survival.

## Data Availability

The current study was based on the results of relevant published studies.

## Abbreviations

PRISMA: Preferred Reporting Items for Systematic Review and Meta-Analysis
CT: Computed tomography
OS: Overall survival
PFS: Progression-free survival
BIA: Bioelectrical analysis
DXA: Dual-energy X-ray absorptiometry
CR-POPF: Clinical related-postoperative pancreatic fistula
PPH: Post-pancreatectomy hemorrhage
DGE: Delayed gastric empty
SSI: Surgical site infection
SMI: Skeletal muscle index
PMI: Psoas muscle index
NOS: Newcastle-Ottawa Scale
HR: Hazard ratio
CIs: Corresponding intervals
OR: Odds ratio
ROC: Receiver operating characteristic
NLR: Neutrophil-lymphocyte ratio
