# Supplementary material for: Association between preoperative sarcopenia and prognosis of pancreatic cancer after curative-intent surgery: a updated systematic review and meta-analysis
Source: World J Surg Oncol. 2024 Jan 30;22:38. doi: 10.1186/s12957-024-03310-y (PMC10825983; doi:10.1186/s12957-024-03310-y)
Supplement: Supplementary file 1 — Additional file 1. [file 12957_2024_3310_MOESM1_ESM.pdf]

Literature search items

Pubmed：

1. [MeSH] pancreatic neoplasms AND [MeSH] sarcopenia

2. [Title/Abstract] (pancreatic cancer OR pancreatic adenocarcinoma OR pancreatic carcinoma OR pancreatic neoplasm) AND (skeletal muscle OR SMI OR psoas muscle OR PMI OR sarcopenia OR body composition)

3. 1 OR 2

Embase：

1. ‘pancreas cancer’/exp AND ‘sarcopenia’/exp

2. 'skeletal muscle':ab,ti OR 'SMI':ab,ti OR ‘psoas muscle’:ab,ti OR ‘PMI’:ab,ti OR ‘sarcopenia’:ab,ti OR ‘body composition’:ab,ti

3. ‘pancreatic cancer’:ab,ti OR ‘pancreatic adenocarcinoma’:ab,ti OR ‘pancreatic carcinoma’:ab,ti OR ‘pancreatic neoplasm’:ab,ti

4. 2 AND 3

5. 1 OR 4

Web of science:

1. TS=’pancreatic neoplasms ‘AND ‘sarcopenia’

2. TI=(pancreatic cancer OR pancreatic adenocarcinoma OR pancreatic carcinoma OR pancreatic neoplasm) AND (skeletal muscle OR SMI OR psoas muscle OR PMI OR sarcopenia OR body composition)

3. 1 OR 2
