# Supplementary material for: Association between preoperative sarcopenia and prognosis of pancreatic cancer after curative-intent surgery: a updated systematic review and meta-analysis
Source: World J Surg Oncol. 2024 Jan 30;22:38. doi: 10.1186/s12957-024-03310-y (PMC10825983; doi:10.1186/s12957-024-03310-y)
Supplement: Supplementary file 2 — Additional file 2: Supplementary Figure 1. Forest plots of comparison between sarcopenia and non-sarcopenia. (A) overall complications, (B) CR-POPF, (C) PPH, (D) DGE, (E) SSI. [file 12957_2024_3310_MOESM2_ESM.pdf]

Supplementary Figure 1. Forest plots of comparison between sarcopenia and non-sarcopenia. (A) overall complications, (B) CR-POPF, (C) PPH, (D) DGE, (E) SSI.

Study or Subgroup	sarcopenia Events	sarcopenia Total	non-sarcopenia Events	non-sarcopenia Total	Weight	Odds Ratio IV, Random, 95% CI	Year
Peng et al. 2012	33	74	115	222	18.2%	0.75 [0.44, 1.27]	2012
Amini et al. 2015	95	192	274	571	21.3%	1.06 [0.77, 1.47]	2015
Wagner et al. 2018	62	145	56	279	19.6%	2.97 [1.91, 4.62]	2018
Choi et al. 2018	34	60	61	120	16.7%	1.26 [0.68, 2.36]	2018
Ratnayake et al. 2019	28	44	29	45	13.0%	0.97 [0.41, 2.30]	2019
Rom et al. 2021	24	30	54	81	11.2%	2.00 [0.73, 5.47]	2021
**Total (95% CI)**		**545**		**1318**	**100.0%**	**1.33 [0.84, 2.12]**	
Total events	276		589				

Heterogeneity: Tau² = 0.23; Chi² = 20.36, df = 5 (P = 0.001); I² = 75%
Test for overall effect: Z = 1.21 (P = 0.23)

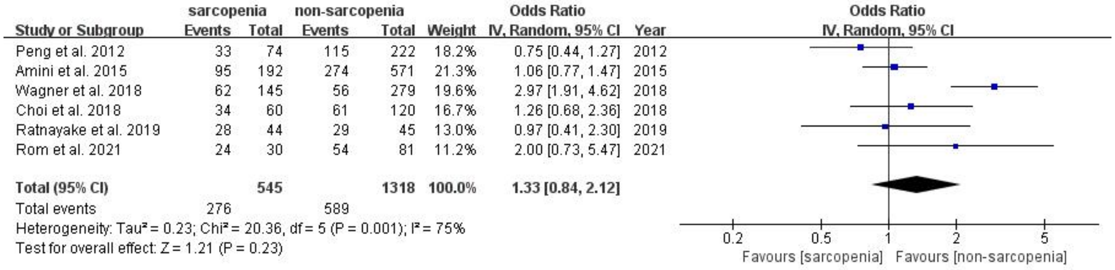

(A)

Study or Subgroup	sarcopenia Events	sarcopenia Total	non-sarcopenia Events	non-sarcopenia Total	Weight	Odds Ratio IV, Random, 95% CI	Year
Nishida et al. 2016	29	132	14	134	16.1%	2.41 [1.21, 4.81]	2016
Takagi et al. 2017	20	55	52	164	17.2%	1.23 [0.65, 2.33]	2017
El Amrani et al. 2018	5	50	4	57	6.6%	1.47 [0.37, 5.81]	2018
Tankel et al. 2018	3	16	12	45	6.3%	0.63 [0.15, 2.62]	2018
Yamane et al. 2018	8	40	22	59	11.4%	0.42 [0.16, 1.07]	2018
Ratnayake et al. 2019	18	44	22	45	13.1%	0.72 [0.31, 1.67]	2019
Gruber et al. 2019	15	78	12	55	12.8%	0.85 [0.36, 2.00]	2019
Ryu et al. 2020	15	252	23	296	16.5%	0.75 [0.38, 1.47]	2020
**Total (95% CI)**		**667**		**855**	**100.0%**	**0.97 [0.65, 1.44]**	
Total events	113		161				

Heterogeneity: Tau² = 0.13; Chi² = 12.04, df = 7 (P = 0.10); I² = 42%
Test for overall effect: Z = 0.17 (P = 0.87)

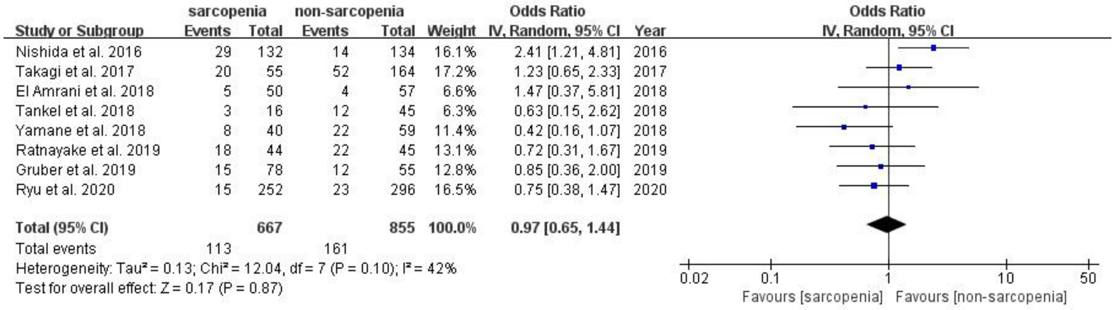

(B)

Study or Subgroup	sarcopenia Events	sarcopenia Total	non-sarcopenia Events	non-sarcopenia Total	Weight	Odds Ratio IV, Random, 95% CI	Year
Ratnayake et al. 2019	3	44	3	45	24.5%	1.02 [0.20, 5.37]	2019
Ryu et al. 2020	8	252	10	296	75.5%	0.94 [0.36, 2.41]	2020
**Total (95% CI)**		**296**		**341**	**100.0%**	**0.96 [0.42, 2.18]**	
Total events	11		13				

Heterogeneity: Tau² = 0.00; Chi² = 0.01, df = 1 (P = 0.93); I² = 0%
Test for overall effect: Z = 0.10 (P = 0.92)

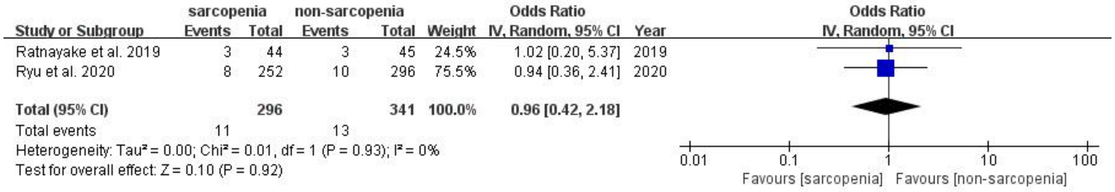

(C)

Study or Subgroup	sarcopenia Events	sarcopenia Total	non-sarcopenia Events	non-sarcopenia Total	Weight	Odds Ratio IV, Random, 95% CI	Year
Nishida et al. 2016	9	132	4	134	13.0%	2.38 [0.71, 7.92]	2016
Takagi et al. 2017	12	55	36	164	20.5%	0.99 [0.47, 2.08]	2017
El Amrani et al. 2018	13	50	17	57	18.4%	0.83 [0.35, 1.93]	2018
Tankel et al. 2018	7	16	8	45	12.4%	3.60 [1.03, 12.54]	2018
Ratnayake et al. 2019	13	44	9	45	16.2%	1.68 [0.63, 4.45]	2019
Ryu et al. 2020	9	252	22	296	19.4%	0.46 [0.21, 1.02]	2020
**Total (95% CI)**		**549**		**741**	**100.0%**	**1.18 [0.67, 2.08]**	
Total events	63		96				

Heterogeneity: Tau² = 0.26; Chi² = 10.83, df = 5 (P = 0.05); I² = 54%
Test for overall effect: Z = 0.58 (P = 0.56)

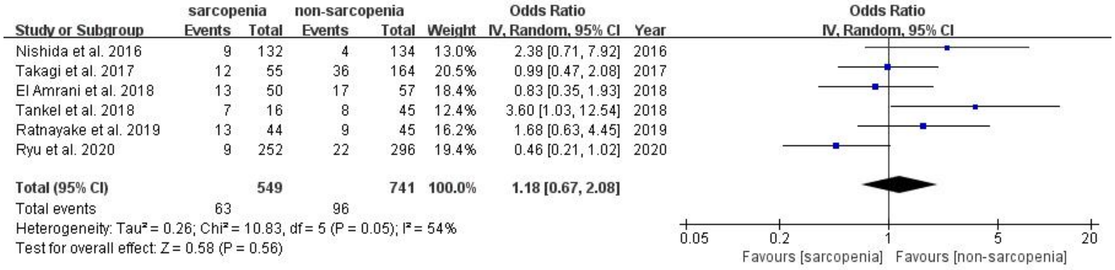

(D)

Study or Subgroup	sarcopenia Events	sarcopenia Total	non-sarcopenia Events	non-sarcopenia Total	Weight	Odds Ratio IV, Random, 95% CI	Year
Nishida et al. 2016	10	132	7	134	16.2%	1.49 [0.55, 4.03]	2016
Takagi et al. 2017	37	55	66	164	23.2%	3.05 [1.60, 5.81]	2017
El Amrani et al. 2018	19	50	19	57	20.0%	1.23 [0.55, 2.71]	2018
Ratnayake et al. 2019	11	44	13	45	17.2%	0.82 [0.32, 2.10]	2019
Ryu et al. 2020	17	252	25	296	23.3%	0.78 [0.41, 1.49]	2020
**Total (95% CI)**		**533**		**696**	**100.0%**	**1.31 [0.75, 2.29]**	
Total events	94		130				

Heterogeneity: Tau² = 0.24; Chi² = 10.10, df = 4 (P = 0.04); I² = 60%
Test for overall effect: Z = 0.96 (P = 0.34)

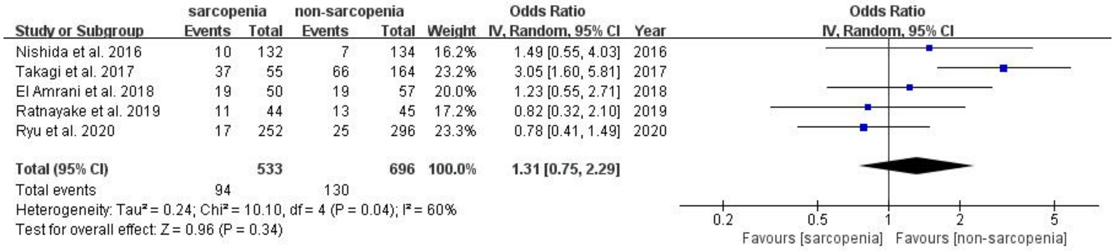

(E)
