# Supplementary material for: Association between preoperative sarcopenia and prognosis of pancreatic cancer after curative-intent surgery: a updated systematic review and meta-analysis
Source: World J Surg Oncol. 2024 Jan 30;22:38. doi: 10.1186/s12957-024-03310-y (PMC10825983; doi:10.1186/s12957-024-03310-y)
Supplement: Supplementary file 3 — Additional file 3: Supplementary Figure 2. Funnel plots for examination of publication bias. (A) overall survival, (B) major complications, (C) profession-free survival. [file 12957_2024_3310_MOESM3_ESM.pdf]

Supplementary Figure 2. Funnel plots for examination of publication bias. (A) overall survival, (B) major complications, (C) profession-free survival

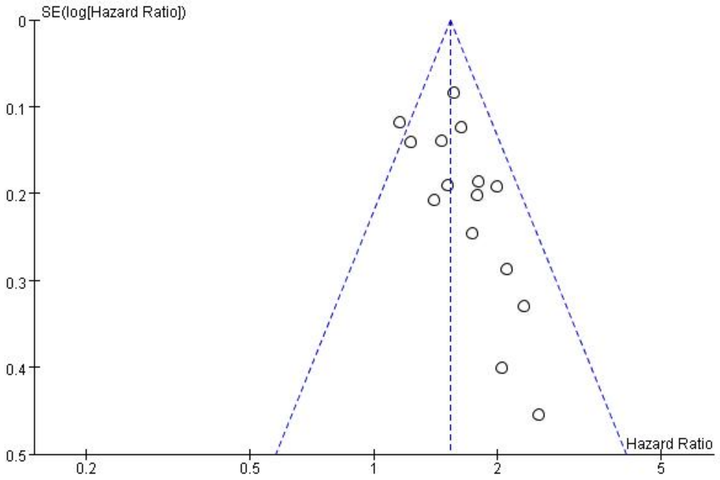

(A)

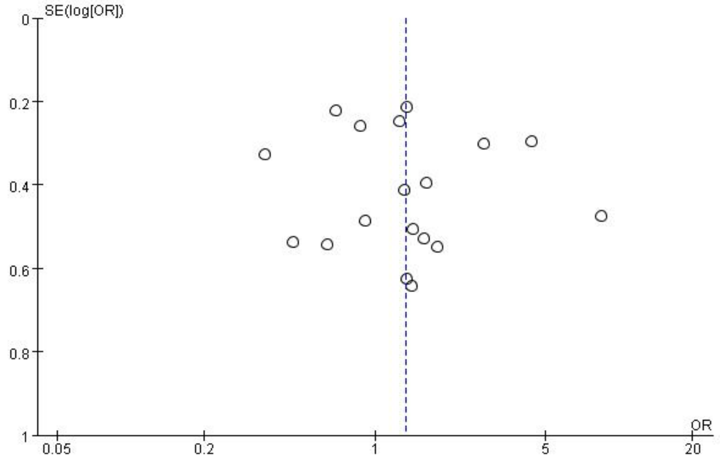

(B)

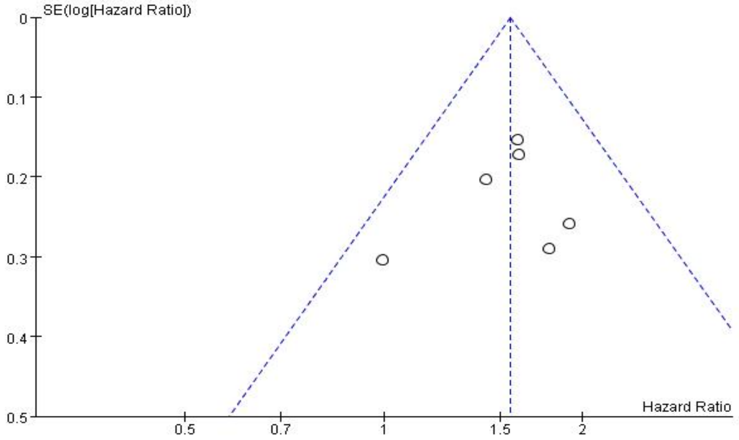

(C)
